# Contrast Sensitivity and Spherical Aberration in Eyes Implanted with AcrySof IQ and AcrySof Natural Intraocular Lens: the Results of a Meta-Analysis

**DOI:** 10.1371/journal.pone.0077860

**Published:** 2013-10-18

**Authors:** Jianping Liu, Jiangyue Zhao, Liwei Ma, Guangcong Liu, Di Wu, Jinsong Zhang

**Affiliations:** 1 Department of Ophthalmology, the Fourth Affiliated Hospital of China Medical University, Shenyang, People’s Republic of China; 2 Eye Hospital of China Medical University, Shenyang, People’s Republic of China; 3 Key Lens Research Laboratory of Liaoning Province, Shenyang, People’s Republic of China; 4 School of Public Health, China Medical University, Shenyang, People’s Republic of China; Massachusetts Eye & Ear Infirmary, Harvard Medical School, United States of America

## Abstract

**Background:**

To systematically evaluate the visual performance of aspheric AcrySof IQ and spherical AcrySof Natural intraocular lens (IOL) after cataract surgery.

**Methodology/Principal Findings:**

Potential randomized controlled trials (RCTs) that involved implanting AcrySof IQ and AcrySof Natural were searched from PubMed, Web of science, EMBASE, Chinese Science and Technology Periodicals Databases and Cochrane Central Register of Controlled Trials. The methodological quality of the studies was assessed by the Jadad method. Standardized mean differences (SMDs) with 95% confidence intervals (CIs) of best-corrected visual acuity (BCVA), contrast sensitivity and spherical aberration were pooled using a random-effects model. Seven studies were identified and analyzed to compare AcrySof IQ (236 eyes) with AcrySof Natural (232 eyes) after phacoemulsification. There was no significant difference in postoperative BCVA between AcrySof IQ and AcrySof Natural (*p* =0.137) after a follow up of 3 months. For contrast sensitivity, these differences reached statistical significance under photopic conditions at two spatial frequencies (3 cycles per degree (cpd), 6 cpd, 12 cpd, and 18 cpd; *p* =0.022, *p* =0.017, *p* = 0.065, and *p*=0.191, respectively) and under mesopic conditions at three spatial frequencies (3 cpd, 6 cpd, 12 cpd, and 18 cpd; *p* =0.007, *p* =0.033, *p* =0.030, and *p* =0.080, respectively). Eyes with AcrySof IQ also had statistically significant less spherical aberration than eyes with AcrySof Natural (*p*<0.001). Sensitivity analysis showed that the results were relatively stable and reliable.

**Conclusions/Significance:**

The overall findings indicate that AcrySof IQ with a modified aspheric surface induced significantly less spherical aberration than AcrySof Natural. Contrast sensitivity in eyes with AcrySof IQ is better than that in eyes with AcrySof Natural, especially under mesopic conditions.

## Introduction

Cataract is the leading reason of blindness in the world, and usually has to be treated surgically [1].

At present, the main surgical procedures are phacoemulsification and intraocular lens (IOL) implantation [2].With the improvement of life quality, cataract surgery has developed from a procedure for the safe removal of the cataract to one aimed at refining to achieve the best possible postoperative refractive result [3]. Although surgical technique as well as IOL materials and designs has improved significantly, there is now an increasing emphasis on improving visual quality and functional vision provided by IOL [4]. Previous studies have demonstrated that contrast sensitivity and wavefront aberration are the potent indicators of functional vision [5,6]. Contrast sensitivity function, measured under varying conditions of luminance and glare, establishes the limits of visual perception across the spectrum of spatial frequencies [7,8]. It determines the relationship between the optical efficiency of the eye and minimal retinal threshold for pattern detection, which is not detected by the measurement of Snellen visual acuity [8].

Spherical aberration is one of the most important higher-order aberrations in the human eye [9].Some scientific studies have shown the decrease in visual quality that occurs with age is usually attributed to lens change. In youth, the generally negative spherical aberration of the crystalline lens largely neutralizes the average positive spherical aberration of the prolate cornea, resulting in an optimized retinal image. In the aging eye, there is a loss of this cornea–lens balancing, resulting in a deterioration of retinal image quality [10-12]. Based on these findings, the AcrySof IQ (model SN60WF, Alcon Surgical Laboratories), is a single-piece foldable acrylic IOL with a posterior aspheric surface (-0.20um, asphericity value) designed to decrease the total amount of ocular spherical aberration after cataract surgery. However, these previous trials generally had small sample sizes and showed conflicting results, which greatly hindered researchers drawing correct conclusions.

Hence, we conducted a meta-analysis of published randomized controlled trials (RCTs) to assess the quality of vision provided by the aspheric IOL (AcrySof IQ) after cataract surgery.

## Materials and Methods

### Literature search strategy

An extensive literature review was searched through PubMed, EMBASE, Chinese Science and Technology Periodicals Databases and Cochrane Controlled Trials Register (most recently updated in 2013 Mar). We performed the literature search with no language limitations. The search terms included were “AcrySof IQ”, “AcrySof Natural”, “comparison”, “contrast sensitivity”, and “spherical aberration”. Abstracts were read and full texts were retrieved if they seemed to meet the objective of this review. Related references and articles were checked and analyzed in depth. Considering all the study design, no previous systematic review or meta-analysis on this topic was found. The study and data accumulation were carried out with approval from the Institutional Review Board of The Fourth Affiliated Hospital of China Medical University and the study complied with the tenets of the Declaration of Helsinki.

### Inclusion Criteria and Exclusion Criteria

The following inclusion criteria were used to identify published studies for this meta-analysis: (1) study design: prospective, randomized clinical controlled trials; (2) population and intervention: patients who were diagnosed as age-related cataract underwent cataract surgery with AcrySof IQ and AcrySof Natural IOL implantation; (3)outcome measurement: variables of the report containing sufficient information on contrast sensitivity and spherical aberration. Exclusion criteria included double reporting, *in vitro* studies, unrelated outcome measurement, use of refractive surgery, delivering no baseline data and no aggregated results. Decisions regarding which trials to be included were made independently by two reviewers (Jianping.Liu, Guangcong Liu). 

### Data extraction

All available data from the selected articles were extracted by two independent reviewers (Jianping Liu, Di Wu) cautiously. The following categories of information were extracted: each study’s author, publication year, study design, study location, number of patients at final follow-up, quality control, duration of follow-up. Whenever any disagreements occurred, they were resolved through discussion till a consensus was made.

### Statistical analysis

 The statistical analysis was carried out through stata software version 10 (StataCorp, College Station, TX). Forest plots were used to present the results, and the results were expressed as standardized mean difference (SMD) and 95% confidence interval (CI). The center of each square indicated the SMD and the size of the square was proportional to percent weight each study contributed to the pooled estimates .The horizonal line bisecting each square represented the 95% CI for the SMD. Heterogeneity among studies was tested using the Chi-squared statistic. If the significant evidence of statistical heterogeneity or clinical diversity was not found (*p* >0.10), fixed-effects model was used [13].However, for the result showing significant heterogeneity (*p* <0.10), we used random-effects model to account for inter-study heterogeneity and tested for statistically significant difference between the estimates with respect to AcrySof IQ and AcrySof Natural IOL. Funnel plot was used to observe the included studies’ publication bias, asymmetry plot implied possible existence of publication bias. The asymmetry degree was measured by Egger’ test, and a *p* value<0.05 was considered as an evidence of publication bias. To explore the steadiness of our results, sensitivity analysis investigating the influence of each individual study on the overall meta-analysis summary estimates was carried out to identify potential outliners [13,14].All statistical tests were two-sided. 

## Results

### Search Results

Initial electronic search retrieved 74 articles after removing duplicates, in which 56 articles were excluded after first-pass review of titles and abstracts. 11 studies were eliminated after full text review according to the inclusion and exclusion criteria specified earlier. Hence a total of 7 prospective RCTs [15-21] were identified. We evaluated the methodological quality of eligible studies using the Jadad method [22]. A checklist contained five items(1): with or without randomization(2); with or without a double-blind design(3); the appropriateness of the randomization methods if used(4); the appropriateness of double-blinding design if used(5); the analysis and reasons for withdrawals and dropouts. Each of the item’s scale is assigned 0 or1.Thus, excellent quality of studies can receive a Jadad score of five, good qualities if the score was 3 or 4, and poor quality if the score was≤2.The characteristics of eligible studies are described in Table 1.

**Table 1:** Characteristics of included studies in the meta-analysis.

Author	Country	Published year	Design	No.of eyes	Time of follow up	Quality assessment (Jadad score)
Awwad ST etal.	USA	2007	RCT	27	3 months	5
				25	3 months	
Nanavaty MA etal.	England	2009	RCT	47	3 months	4
				47	3 months	
Chen YY etal.	China	2012	RCT	28	3 months	3
				27	3 months	
ROCHA KM etal.	Brazil	2006	RCT	40	3 months	4
				40	3 months	
Tzelikis PF etal.	Brazil	2007	RCT	25	3 months	4
				25	3 months	
Pandita D etal.	India	2007	RCT	36	3 months	4
				37	3 months	
Li HW etal.	China	2009	RCT	33	3 months	3
				31	3 months	

RCT,randomized controlled trial.

### Meta-Analysis Results

**Figure 1:**
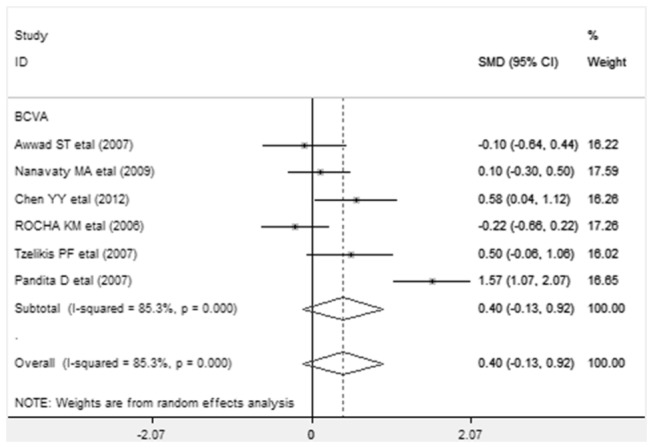
Standardized mean difference (SMD) in best-corrected visual acuity (BCVA): This forest plot showed the SMD in BCVA (log MAR) along with associated 95% confidence interval (CI), comparing AcrySof IQ and AcrySof Natural intraocular lens.

Differences in mean changes in best-corrected visual acuity (BCVA) after implantation, along with SMD and 95% CI, are presented in Figure 1.The analysis results of BCVA of each study were presented in a forest plot. Values to the left of the vertical line at 0 show greater change in the subjects implanted with AcrySof Natural and values to the right of the vertical line show greater change in AcrySof IQ. The subtotal rows show the meta-analysis summary values for each time point. No significant difference in BCVA was found in AcrySof IQ group compared with AcrySof Natural group (SMD=0.399, 95%CI-0.127 to 0.924, *p* =0.137). 

**Figure 2:**
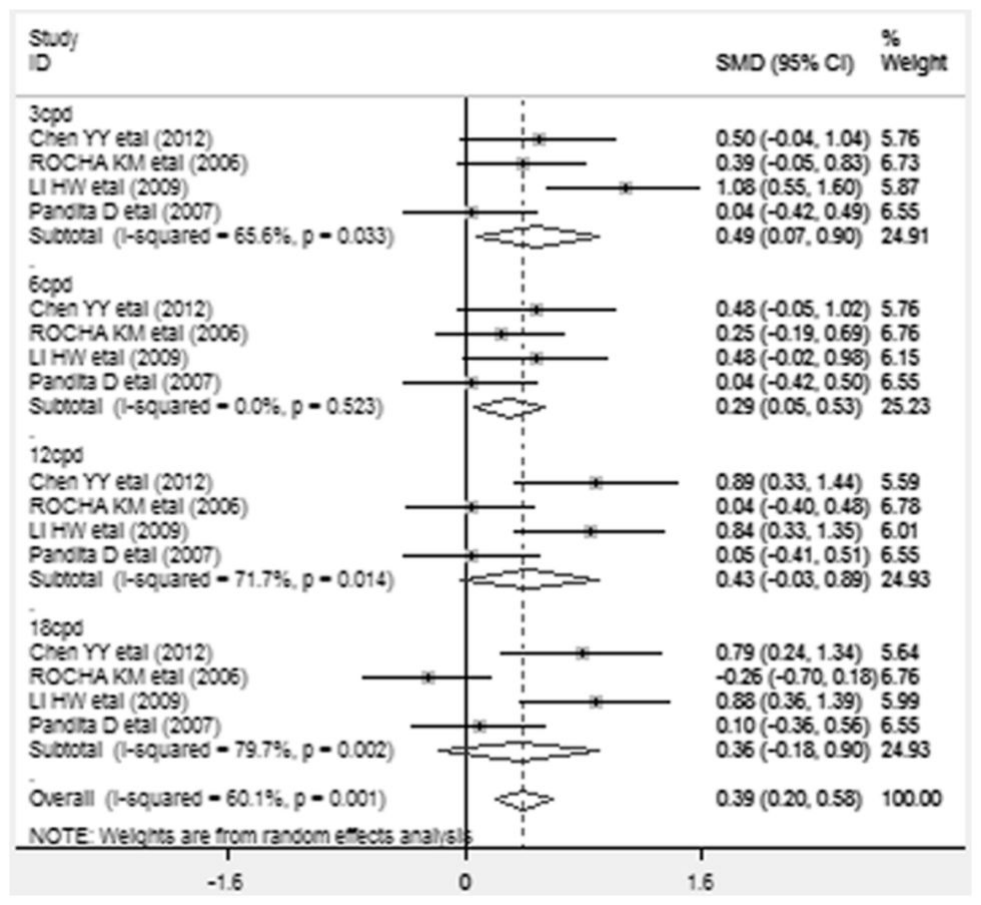
Standardized mean difference (SMD) in contrast sensitivity: This forest plot showed the SMD in postoperative contrast sensitivity under photopic conditions at 3 cycles per degree (cpd), 6 cpd, 12 cpd, and 18 cpd along with associated 95% confidence interval (CI), comparing AcrySof IQ and AcrySof Natural intraocular lens.

**Figure 3:**
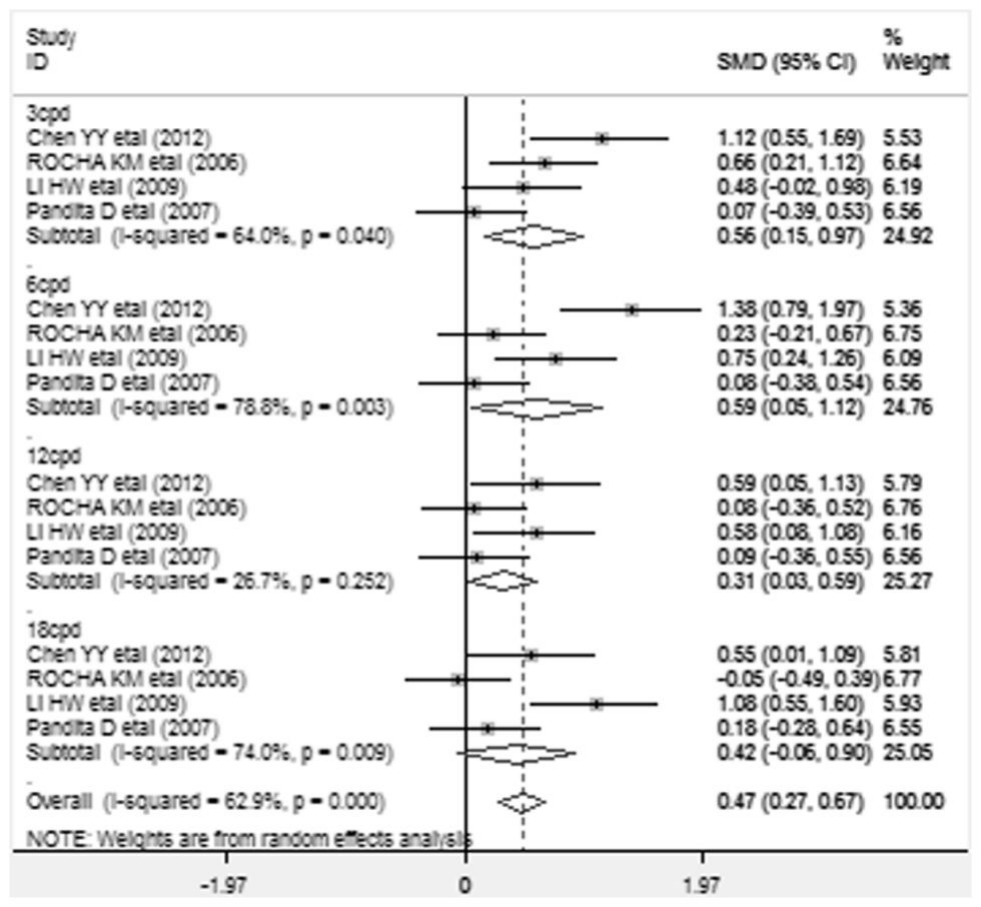
Standardized mean difference (SMD) in contrast sensitivity: This forest plot showed the SMD in postoperative contrast sensitivity under mesopic conditions at 3 cycles per degree ( cpd ), 6 cpd, 12 cpd, and 18 cpd along with associated 95% confidence interval (CI), comparing AcrySof IQ and AcrySof Natural intraocular lens.

Changes in contrast sensitivity were assessed under photopic and mesopic conditions at low spatial frequency (3 cycles per degree [cpd]), intermediate frequency (6 cpd), high frequencies (12 cpd and 18 cpd). Differences in mean changes in contrast sensitivity after implantation, along with SMD and 95% CI are presented. The analysis results of contrast sensitivity of each study were presented in a forest plot. Values to the left of the vertical line at 0 show greater change in contrast sensitivity in the subjects implanted with AcrySof Natural and values to the right of the vertical line show greater change in AcrySof IQ. Significant improvements in contrast sensitivity under photopic conditions(Figure 2) at 3cpd,6cpd spatial frequencies (SMD=0.486,95%CI 0.069 to 0.903, *p* = 0.022;SMD=0.291,95%CI 0.052 to 0.531, *p* = 0.017) and under mesopic conditions (Figure 3) at 3cpd,6cpd ,12cpd spatial frequencies (SMD=0.564, 95%CI 0.155 to 0.973, *p* =0.007;SMD=0.585,95%CI 0.048 to1.122, *p* =0.033; SMD=0.311, 95%CI 0.030 to 0.592, *p* =0.030) were found in AcrySof IQ group compared with AcrySof Natural group. There were no statistically significant differences in contrast sensitivity between AcrySof IQ and AcrySof Natural in photopic conditions at 12cpd, 18cpd (SMD =0.433, 95% CI -0.026 to 0.892, *p* = 0.065; SMD=0.362, 95% CI -0.181 to 0.904, *p* = 0.191) and mesopic conditions at 18 cpd (SMD=0.423, 95%CI -0.056 to 0.902, *p* =0.423).

**Figure 4:**
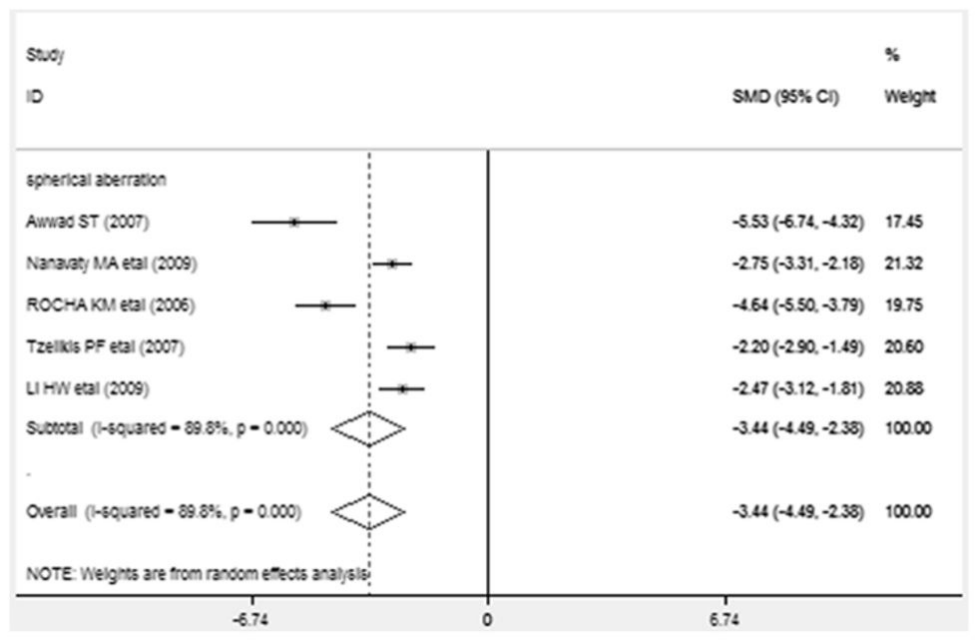
Standardized mean difference (SMD) in spherical aberration: This forest plot showed the SMD in spherical aberration along with associated 95% confidence interval (CI), comparing AcrySof IQ and AcrySof Natural intraocular lens.

Differences in mean changes in spherical aberration after implantation, along with SMD and 95% CI are presented in Figure 4. The analysis results of spherical aberration of each study were presented in a forest plot. Values to the left of the vertical line at 0 show greater change in spherical aberration in the subjects implanted with AcrySof IQ and values to the right of the vertical line show greater change in AcrySof Natural. Significant improvement in spherical aberration was found in the AcrySof IQ group compared with AcrySof Natural group (SMD=-3.435, 95% CI-4.487 to -2.384, *p* <0.001).

Sensitivity analysis was carried out by removal of one RCT at a time and computing the pooled SMDs for the remaining studies. We found no individual study could alter the pooled results of BCVA (SMD = 0.399, 95% CI -0.09 to 0.73, *p* =0.137) ,contrast sensitivity under photopic conditions (SMD= 0.389, 95%CI 0.19 to 0.53, *p* <0.001) and mesopic conditions (SMD= 0.467,95CI%0.26 to 0.59, *p* <0.001) as well as spherical aberration (SMD=-3.435, 95% CI -3.62 to -2.41, *p* <0.001),which indicated that our results were stable and reliable.

### Publication bias

**Figure 5:**
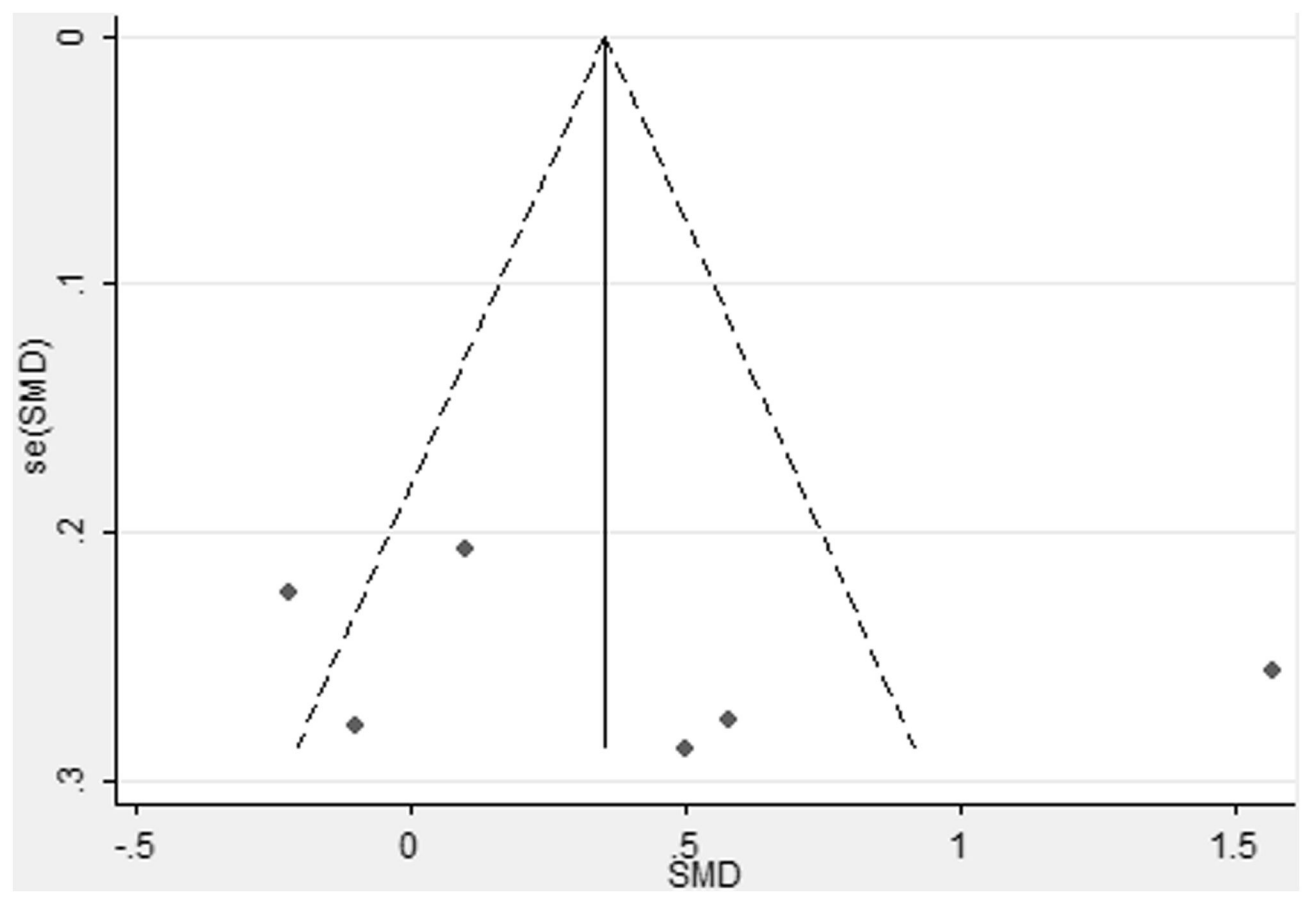
Funnel plot for the results between AcrySof IQ and AcrySof Natural intraocular lens. Egger’ publication bias test result (*p* =0.476).

Funnel plot was performed to assess the publication bias of literatures (Figure 5). There was low possibility of asymmetry in the funnel plot because a limited number of trials were involved in the final analysis, but the result of Egger’s test did not show any evidence of publication bias (t=0.79, *p* = 0.476). 

## Discussion

Focus on the quality of life today, with advances in cataract surgery and IOL implantation, the visual outcome is measured not only by means of visual acuity, but also by quality of vision [23]. Contrast sensitivity and wavefront aberration are the main characteristics of the quality of vision [24,25]. Our study evaluated the visual outcomes between aspheric IOL (AcrySof IQ) and spherical IOL (AcrySof Natural) implantation. In this meta-analysis, we identified a number of relevant studies (n=7), and it should be noted that all of the studies did randomize treatments, which could be considered as sufficient evidence reporting.

Currently, evidence of vision quality based on implanting AcrySof IQ and AcrySof Natural IOLremains lacking. Despite a prospective RCT of AcrySof IQ and AcrySof Natural implants might go some way to provide the answer to the question. However, such a trial would require a great deal of patients to provide conclusive result. So we carried out the meta-analysis to evaluate the visual outcomes of AcrySof IQ IOL. Meta-analysis is a method of using statistical analysis that combines or integrates the results of several independent studies considered by the analyst to be combinable [13,26]. By combining the results of these RCTs through the meta-analysis, our study has a greater statistical power than the powers of the studies as individuals. Based on our result, there was no clinically relevant difference in BCVA (logMAR) between AcrySof IQ and AcrySof Natural implantation, which indicates that measuring visual acuity at different contrast levels is a more sensitive measure as it offers the various visual cues of daily life and may display more preferable information about functional vision than the information provided by the high contrast letters of Snellen acuity charts [19].

Cataract surgery is becoming more of a refractive procedure, patients who acquire good vision after surgery, at the same time, parts of the sufferings have appeared the phenomenon of glare, halos, and poor night vision [27,28]. Improvement in contrast sensitivity could influence the quality of vision. Measuring contrast sensitivity under different luminance and glare conditions is a more sensitive measure as it gives much better perception of visual status and contrast testing determines contrast thresholds at each spatial frequency in normal eyes as well as in eyes with ocular disease [29,30]. Traditional AcrySof Natural IOL that is designed without prolate surface modification decreases the optical transfer function after cataract surgery, which is leading to a reduction in the quality of the retinal image. In a prospective study by Kennis et al [31], contrast sensitivity was significantly better at almost all spatial frequencies in the Tecnis Z9000 compared with Sensar AR40e, or AcrySof Natural SN60AT. Contrary to the findings of Kennis et al, a study by Mun˜oz [32], spherical aberration and contrast sensitivity with the Tecnis Z9000 IOL, found no statistically significant differences between the AR40e IOL and Z9000 IOL in photopic and mesopic contrast sensitivity.

With the development of wavefront aberration techniques, in the design of IOLs, an attempt has been made to mirror natural crystal as closely as possible [33]. Spherical aberration is a property of all spherical IOLs, which is one of the most important aberrations contributing to visual deterioration of the pseudophakic eye [34,35]. It occurs when the lens bends peripheral rays more strongly (positive spherical aberrations). The flatter curve of the peripheral cornea refracts light less strongly than the steeper central area [36]. Conventional spherical IOLs can result in positive spherical aberration and are unable to offset corneal positive spherical aberration, ultimately leading to a decline in image quality. 

Wang et al [37] conducted a meta-analysis to compare the visual performance of aspheric IOL with spherical IOL, but IOLs made from different materials and optical designs may have different optical properties, which is difficult to get a conclusive recommendation in the selection of a particular IOL for an individual [38]. In our meta-analysis, the intraindividual comparative study was implanted with aspheric IOL (AcrySof IQ) and the design-equivalent spherical IOL (AcrySof Natural).The results showed that AcrySof IQ provided significant improvement in contrast sensitivity under mesopic conditions at almost all spatial frequencies. We found a statistically significant decrease in spherical aberration between AcrySof IQ group and AcrySof Natural group. 

There were some limitations to this study: (1) Only a small number of trials were enrolled in this meta-analysis, (2) There was a follow up of 3 months. It may be that different measurements are obtained in a longer term study at least one year, and (3) We only evaluated spherical aberration, which might not represent the visual performance of total higher-order aberrations.

In summary, findings of the present study indicate that visual quality can be improved by implantation of an IOL with a modified prolate surface. Patients will be more generally satisfied after implantation of an AcrySof IQ IOL because they will have less spherical aberration and better contrast sensitivity, especially under dim light. Future multi-center RCTs of AcrySof IQ versus AcrySof Natural IOL are warranted for further research and development.

## Supporting Information

Figure S1 **PRISMA 2009 Flow Diagram.**
(DOC)

Checklist S1 **PRISMA 2009 Checklist.**
(DOC)
