# Supplementary material for: Contrast Sensitivity and Spherical Aberration in Eyes Implanted with AcrySof IQ and AcrySof Natural Intraocular Lens: the Results of a Meta-Analysis
Source: PLoS One. 2013 Oct 18;8(10):e77860. doi: 10.1371/journal.pone.0077860 (PMC3799620; doi:10.1371/journal.pone.0077860)
Supplement: Figure S1 — PRISMA 2009 Flow Diagram. (DOC) [file pone.0077860.s001.doc]

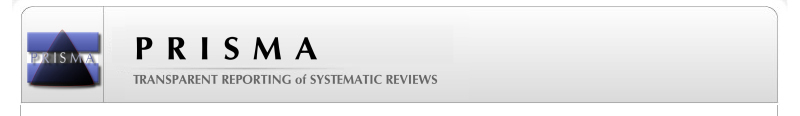
PRISMA 2009 Flow Diagram

**Screening**

**Included**

**Eligibility**

**Identification**

Records identified through database searching
(n =106)

Additional records identified through other sources
(n=0)

Records after duplicates removed
(n=74)

Records screened
(n=74)

Records excluded
(n=56)

Full-text articles assessed for eligibility
(n=18)

11 were excluded:(1) unmet inclusion criterion n=7;(2) no baseline data n=2;(3)duplicate n=2

Studies included in qualitative synthesis
(n=7)

Studies included in quantitative synthesis (meta-analysis)
(n=7)
